# A Participatory Approach to Designing and Enhancing Integrated Health Information Technology Systems for Veterans: Protocol

**DOI:** 10.2196/resprot.3815

**Published:** 2015-02-27

**Authors:** Jolie N Haun, Kim M Nazi, Margeaux Chavez, Jason D Lind, Nicole Antinori, Robert M Gosline, Tracey L Martin

**Affiliations:** ^1^HSR&D Center of Innovation on Disability and Rehabilitation ResearchJames A. Haley Veterans HospitalTampa, FLUnited States; ^2^Veterans and Consumers Health Informatics OfficeVeterans Health AdministrationDepartment of Veterans AffairsAlbany, NYUnited States; ^3^Department of Veterans AffairsJames A. Haley Veterans HospitalTampa, FLUnited States; ^4^Department of Veterans AffairsVA New England Health Care SystemBedford, MAUnited States

**Keywords:** veterans, patient-provider communication, Department of Veterans Affairs, mixed methods, patient-centered care

## Abstract

**Background:**

The Department of Veterans Affairs (VA) has developed health information technologies (HIT) and resources to improve veteran access to health care programs and services, and to support a patient-centered approach to health care delivery. To improve VA HIT access and meaningful use by veterans, it is necessary to understand their preferences for interacting with various HIT resources to accomplish health management related tasks and to exchange information.

**Objective:**

The objective of this paper was to describe a novel protocol for: (1) developing a HIT Digital Health Matrix Model; (2) conducting an Analytic Hierarchy Process called pairwise comparison to understand how and why veterans want to use electronic health resources to complete tasks related to health management; and (3) developing visual modeling simulations that depict veterans’ preferences for using VA HIT to manage their health conditions and exchange health information.

**Methods:**

The study uses participatory research methods to understand how veterans prefer to use VA HIT to accomplish health management tasks within a given context, and how they would like to interact with HIT interfaces (eg, look, feel, and function) in the future. This study includes two rounds of veteran focus groups with self-administered surveys and visual modeling simulation techniques. This study will also convene an expert panel to assist in the development of a VA HIT Digital Health Matrix Model, so that both expert panel members and veteran participants can complete an Analytic Hierarchy Process, pairwise comparisons to evaluate and rank the applicability of electronic health resources for a series of health management tasks.

**Results:**

This protocol describes the iterative, participatory, and patient-centered process for: (1) developing a VA HIT Digital Health Matrix Model that outlines current VA patient-facing platforms available to veterans, describing their features and relevant contexts for use; and (2) developing visual model simulations based on direct veteran feedback that depict patient preferences for enhancing the synchronization, integration, and standardization of VA patient-facing platforms. Focus group topics include current uses, preferences, facilitators, and barriers to using electronic health resources; recommendations for synchronizing, integrating, and standardizing VA HIT; and preferences on data sharing and delegation within the VA system.

**Conclusions:**

This work highlights the practical, technological, and personal factors that facilitate and inhibit use of current VA HIT, and informs an integrated system redesign. The Digital Health Matrix Model and visual modeling simulations use knowledge of veteran preferences and experiences to directly inform enhancements to VA HIT and provide a more holistic and integrated user experience. These efforts are designed to support the adoption and sustained use of VA HIT to support patient self-management and clinical care coordination in ways that are directly aligned with veteran preferences.

## Introduction

### Health Information Technology in the Veterans Health Administration

Historically, health information technology (HIT) applications and systems have been developed under the auspices of independent organizational program offices, and as a result, they may not optimally support or enable an integrated patient experience across technology platforms [1]. Monolithic systems that lack an integrated strategy can result in a fragmented user experience and lead to system and resource inefficiencies [2-4]. In contrast, the development and implementation of a comprehensive and integrated approach to HIT, based on patient preferences and goals in various contexts, can have meaningful effects on patient engagement, empowerment, quality of care, and health outcomes [5,6].

To enable a more patient-centered and integrated approach to HIT tools and services, the Department of Veterans Affairs (VA) chartered a Connected Health task force in 2012 to develop strategic recommendations that would enable a seamless, unified veteran experience across all VA sponsored patient-facing technologies (ie, any technologies that a patient uses directly). Task force recommendations included the development of a centralized governance structure that would integrate and standardize the development and deployment of VA digital health tools and services. The Office of Connected Health of the Veterans Health Administration (VHA) was established in 2013, and represents a centralized governance model for multiple VHA program offices responsible for patient-facing technology systems, including My Health***e***Vet, Web and Mobile Solutions, and the VHA Innovation Program [7]. The Office of Connected Health is part of the VA Central Office organizational structure and is responsible for aligning virtual care technologies.

As emphasized in the VA Strategic Plan [8], veterans need an integrated system of HIT resources and tools that are useful and easy to use, so they can take an active role in their health care management. Increasingly, these HIT tools must support virtual care, while also connecting with VA enterprise-wide clinical information systems,

The development and proliferation of virtual access to care supports an organizational approach that is personalized, proactive, and patient-driven...Advances in virtual care expand where health care services can be accessed, reduce the need for travel to medical facilities, and transform VA’s delivery of health care and its effect on patients’ health outcomes. (pg. 18). [8]

To date, the VA has invested in a multitude of electronic health resources, such as Telehealth, VetLink Kiosks, an electronic health record, a tethered patient portal known as My Health***e***Vet, and mobile-based applications to increase patient access, support self-management, enhance patient-provider communication, and improve patient health outcomes. As noted by the task force, however, further integration and alignment of these HIT resources is needed to provide a consistent and optimal user experience (including common user interfaces and standardized information displays) that is based on veterans’ needs and preferences.

### Veterans Preferences for Health Information Technology Resources

To accomplish this effectively, it is crucial to understand the nuances of veteran preferences for using various types of HIT resources to accomplish common user tasks and to exchange information. Concurrently, a detailed assessment of current and future VA patient-facing technology platforms is needed in order to identify integrated approaches that will best support a more patient-centered experience. In alignment with the Office of Connected Health, this VA funded research is designed to: (1) conduct a comprehensive assessment of current and future patient-facing technology resources based on the input of an expert panel that includes organizational subject matter experts and key stakeholders; (2) learn which resources veterans prefer to use to accomplish their health related tasks within a given context; (3) identify veteran preferences for using VA resources to exchange information; and (4) explore how veterans want to interact with digital health resource interfaces (eg, look, feel, and function). The study aims are designed to support veterans’ self-management and task accomplishment (eg, accessing lab test results, refilling a prescription, scheduling an appointment, communicating electronically, etc), and to improve continuity of care through the integrated use of the VA’s electronic health resources. Specifically they are to: (1) explore the preferences of veterans with chronic comorbid conditions for using various VA electronic health resources, using participatory research methods (develop and refine visual modeling simulations of VA electronic resources based on direct veteran input); (2) develop a comprehensive Digital Health Matrix Model of current and future VA patient-facing electronic health resources and conduct an Analytical Hierarchy Process, a pairwise comparison process with expert panel members and veteran participants; and (3) collaborate with VHA Program Offices as operational partners to directly inform current and future patient-facing HIT redesign efforts.

In this paper, we describe our development processes and study protocol, which leverage stakeholder groups (subject matter experts, clinicians, and veterans) and a participatory research approach that purposively recruits participants as expert informants to express their preferred vision for the future of VA’s system of electronic health resources. Products of this research will be used in tandem with VA operational efforts to increase the usability and usefulness of the VA’s electronic health resources, and to support a more integrated and effective veteran experience in use of VA HIT electronic health resources.

## Methods

### Study Design Overview

This mixed-methods participatory descriptive study [9] collects data using both an expert panel (organizational subject matter experts, clinicians, and operational stakeholders) and a series of veteran focus groups. The expert panel constructs a Digital Health Matrix and subsequently analyzes it using a structured pairwise comparison process, based on Analytical Hierarchy Process techniques (see the Data Collection section) [10]. The purpose of the Digital Health Matrix Model will be two-fold, first, to create a novel comprehensive descriptive inventory of VA’s electronic health resources, and second, to provide an informational tool which will enable expert panel members to complete pairwise comparisons of various electronic health resources.

To complete data collection, two rounds of veteran participant focus groups will be conducted, along with self-administered surveys to elicit veteran perspectives. Based on the first round of veteran focus groups, the research team will collaborate with members of the VHA Human Factors team to develop relevant process models, and then create a set of visual modeling simulations. A second round of focus groups with the same veterans from the first round will then be conducted to elicit feedback about the visual modeling simulations, and to complete pairwise comparisons adapted to the focus group format. These focus groups will contribute to a final set of visual modeling simulations and a refined Digital Health Matrix Model. This study is approved and regulated by the VA Central Institutional Review Board.

### Sample and Sampling

In qualitative research, sample size relies on the quality and richness of information obtained [11,12]. Achieving conceptual saturation is the goal of qualitative research, and is not dependent on sample size, but on the ability of the data to support interpretations, for example theoretical saturation [11,12]. Furthermore, recruiting the right participants is critical to gaining the most valuable information to articulate an integrated vision for VA electronic health resources. We will conduct our research with two primary groups: (1) an expert panel; and (2) veterans. These sample groups are described in further detail in the following paragraphs.

### Expert Panel

Organizational subject matter experts and key stakeholders will comprise the expert panel. This panel will include representation from all relevant VHA Program Offices and key clinical disciplines. The majority of these individuals are well known by the research team as key representatives for each of VA’s electronic health resource platforms, clinicians, subject matter experts who work in this area of research, and Office of Connected Health representatives.

Snowball sampling will be used as needed to recruit members that represent all key stakeholders (eg, My Health***e***Vet, Telehealth, Mobile Health, Vetlink Kiosks, phone/texting, clinicians, patient educators, etc). Potential expert panel members will be invited to participate via email. To ensure robust input, expert panel members will also be invited to assess any gaps in representation, and nominate other experts or stakeholders to participate. This process will continue until all stakeholder groups are well represented. As indicated by VHA regulations, panel members are participating as employees during their regular work schedule, and thus will not receive compensation for their participation.

### Veteran Sample

The research team is purposively recruiting up to N=48 veterans who have expressed interest in using HIT electronic resources as “expert informants” to inform the outcomes of this project. The sample will include English speaking veterans age 35 years and older, with at least two chronic comorbid conditions (eg, diabetes and high blood pressure), and who report using two or more VA electronic health resources or non-VA electronic resources more than once a month. Therefore, study exclusion criteria include veterans younger than 35 years of age with less than two comorbid conditions, who use fewer than two VA electronic health resources less than once a month. Because of the nature of this study, we exclude those who do not speak English; and/or who report a visual, hearing, or cognitive impairment. This part of the study is being conducted at two VA Medical Centers: (1) the James A Haley Veterans Hospital (Tampa, Florida); and (2) the VA New England Health Care System, (Bedford, Massachusetts).

**Figure 1:**
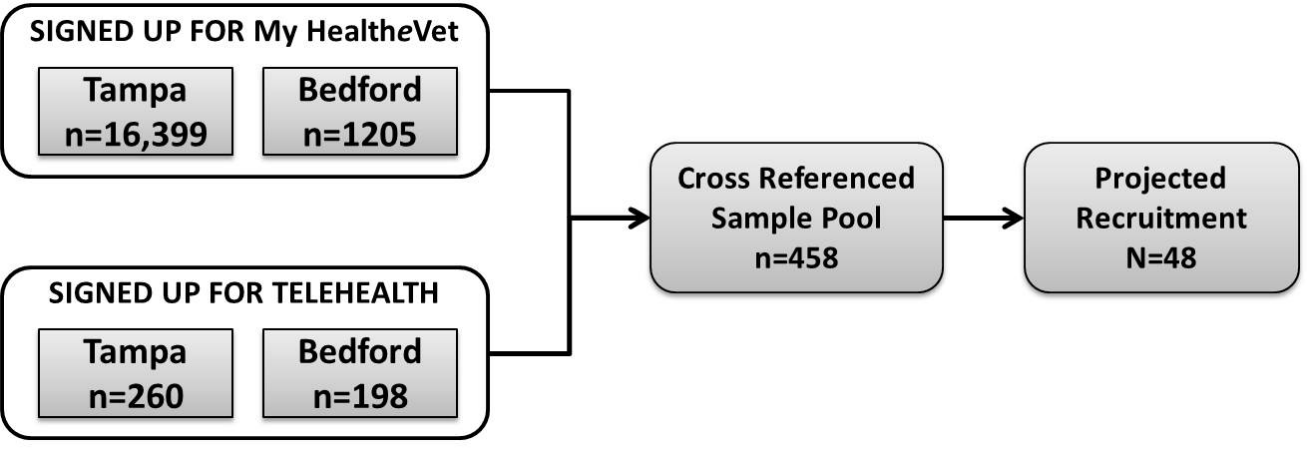
Veteran participant recruitment flow chart.

Purposive sampling will yield a sample pool for veteran recruitment efforts. We used administrative data to identify veterans registered for My Health***e***Vet, who also completed the in-person process of authenticating their identity and opted in to use Secure Messaging. This approach identified 16,399 potential participants at Tampa; and 1205 potential participants at Bedford. Next we cross-analyzed the list of potential participants to identify veterans who have also used VA Telehealth services to ensure that study participants had access to use at least two forms of VA electronic health resources. In this process, we identified 260 potential participants in Tampa and 198 in Bedford. All 458 potential participants will be contacted and screened using a structured questionnaire to ensure information rich sources. We aim to recruit approximately 10% of the sample pool (48 participants), depending upon when theoretical saturation is reached. Figure 1 shows the recruitment process for veteran focus groups.

A structured screening interview tool was developed that includes items to address study age criteria and the occurrence of at least two chronic comorbid conditions (eg, diabetes, high blood pressure, COPD, etc). Based on Agency for Healthcare Research and Quality (AHRQ) recommendations, the screening interview tool also includes items to ensure veteran use of at least two VA electronic health resources (to make transactions; and access, store, manage, organize, and track information) [13]. This process of purposive sampling will ensure recruitment of individuals who already use available VA electronic health resources, and who may also utilize non-VA electronic resources. The screening interview tool also includes items to assess use of specific VA HIT resources, (including My Health***e***Vet, Kiosks, mobile applications, Telehealth, etc), and to identify any visual, hearing, or cognitive impairment.

Study team members will contact potential study participants for recruitment via telephone utilizing the screening tool until domain and theme saturation is reached in data collection. Veterans will receive up to US $50 for their participation (US $25 for participating in each round of focus groups).

### Data Collection

**Figure 2:**
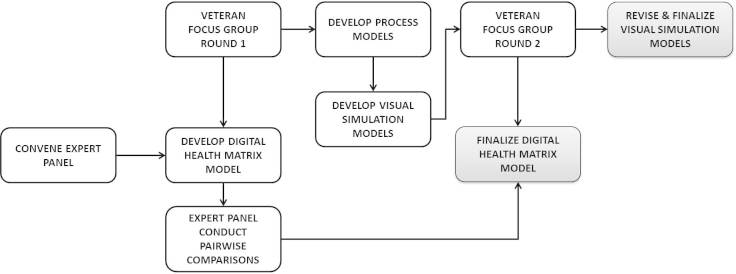
Study flow chart.

Data will be collected from expert panel members using a series of teleconference calls to inform the development of the Digital Health Matrix Model, and communication via email to request and obtain individual responses for a structured pairwise comparison activity that is based on Analytical Hierarchy Process techniques. Expert panel members will also be invited to participate in an education session via teleconference that provides additional instructions on completing the requested pairwise comparisons. Data will be collected from veteran participants using a self-administered survey and focus group scripts. Figure 2 shows the study data collection process.

### Digital Health Matrix Model and Analytical Hierarchy Process Pairwise Comparisons

Pairwise comparison is an analytic hierarchy process (ie, method for understanding complex decision making) that directs participants to compare a series of items and decide which item is preferred. Participants then quantify their preferences using a numerical scale [10]. For the purpose of this study, veterans and expert panel members will use information provided by the Digital Health Matrix to compare and prioritize VA HIT and other VA and non-VA electronic resources. Pairwise comparisons will be conducted in four steps [10]. The first step is defining the problem, for example, what patient-facing electronic health resources are available to veterans, and what are the resource features and elements for prioritization. The research study team has identified relevant VA electronic health resource platforms (eg, My Health***e***Vet, Mobile Health, VetLink Kiosks, Telehealth, etc), features (eg, Secure Messaging, Blue Button, Prescription Refill, etc), and elements for prioritization (eg, access/availability, specific resources, user groups, and context). This initial activity facilitates a focus on available electronic health resources, and their functions and features. This preliminary content will be revised throughout the process, particularly as data are collected from expert panel members and veterans in subsequent steps of the process.

The second step entails structuring the decision hierarchy and emphasizes expanding content developed in the first step through an information gathering process with subject matter experts, stakeholders, and veteran input. To complete this second step, we will develop a Digital Health Matrix Model that represents a detailed inventory of available electronic resources, their features, characteristics, and contexts for use.

To develop the Digital Health Matrix Model, an expert panel will provide the appropriate clinical, administrative, and operational expertise and perspective. Expert panel stakeholder groups include clinicians (physicians, nurses, and patient educators) and representatives aligned with each of the VA electronic health resources (My My Health***e***Vet, Telehealth, Mobile Health, VetLink Kiosks, etc), veterans who participate in the focus groups will also provide additional input to inform the development and refinement of the matrix, especially during the first round of veteran focus groups.

The multi-axis Digital Health Matrix Model will include both currently available and future VA patient-facing platforms, their features, their availability, and conditions for appropriate use. Due to the complexity of VA electronic health resources and elements of interest, the Digital Health Matrix Model will be developed using an electronic Excel document with several sheets representing topics discussed during focus group interviews such as access, function, preferences, barriers to use, relevant user tasks, etc. Within each sheet, there will be a two-axis inventory of: (1) each electronic health resource (represented by row); and (2) domains (ie, broad categories describing related items) that emerge from focus group data and are relevant to the topic of each sheet. This matrix model will provide a descriptive blueprint for veteran decision making, and support the continued development of a more integrated system of VA patient-facing platforms and electronic health resources that meet the needs and preferences of veteran users. The organization of the matrix document will also allow content to be evaluated and prioritized based on the perceived usefulness of each electronic health resource within specific contexts. Due to the length and breadth of detail contained within the model, we will develop search and categorization options to allow expert panel members to easily select and compare two (or more) resources while completing the pairwise comparison activity.

Both expert panel members and veterans will conduct a single activity that will complete the third and fourth steps of the process. There are two separate processes that will be used to complete these activities with the panel members and veterans. Using email, expert panel members will be provided a series of “worksheets” in a single document, with each worksheet representing a single health management task. Panel members will be asked to consider veterans preferences, cost, convenience, and workflow consequences, when completing the pairwise comparisons to determine which electronic health resources most effectively support health care management within the specific, predefined contexts. An adapted process will be completed by veterans during the second round of focus groups to ensure that their input is represented in the final model. Veterans will complete the pairwise comparisons and rank their preferences as a group to promote discussion.

**Figure 3:**
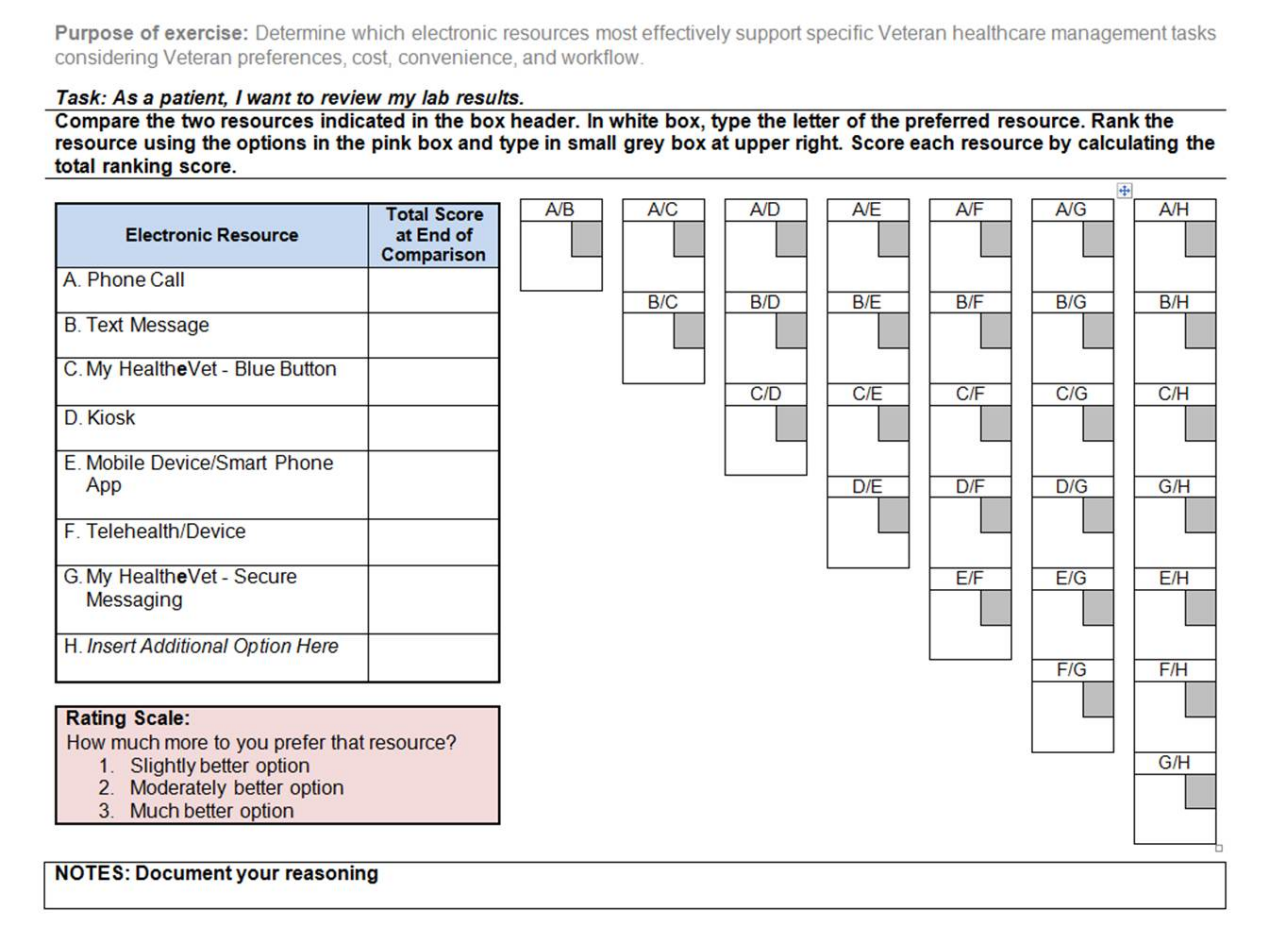
Pairwise comparison worksheet example.

In these final steps, electronic resources are compared (step 3) and ranked (step 4). Comparisons in step 3, allow the individual(s) to consider the value of using electronic health resources for a given health management task. In step 4, the selected electronic health resources are ranked to determine their level of priority over other options; numerical priorities are assigned on a scale of 1 to 3. This is done to calculate numeric weights for each alternative. The final scores provide a decision-making model that compares alternative electronic health resources for accomplishing specific tasks for managing a health condition. Figure 3 shows an example of a pairwise comparison worksheet for a single task.

### Veteran Demographic Survey and Assessments

As previously noted, veteran participants will participate in two rounds of focus groups. At the first round of focus groups, veteran participants (up to N=48) will be consented and complete a baseline participant survey and assessment packet with a research team member in a private room. To collect demographic data, they will complete a 9-item demographic survey to ascertain age/date of birth, gender, race/ethnicity, education level, income level, marital status, and current medical conditions. There are thirty additional items that will be included to assess their electronic resource use (such as use of computer, Internet, smart phone, mobile technology, etc) and use of VA specific HIT resources such as My Heath*e*Vet, Secure Messaging, etc. Health literacy will be assessed using two validated instruments: (1) the BRIEF health literacy screening, and (2) the Rapid Estimate of Adult Literacy in Medicine (REALM) survey. The BRIEF is a 4-item self-report screening tool to assess health literacy skills [14]. The REALM assesses health literacy by having respondents verbally articulate three columns of twenty-two health related terms [15]. Electronic health literacy will also assessed using two instruments: (1) the eHealth Literacy Scale (eHEALS), and (2) the Computer-Email-Web (CEW) Fluency Scale. The eHEALS is a 10-item measure of eHealth literacy developed to measure consumers' knowledge, comfort, and perceived skills at finding, evaluating, and applying electronic health information to health problems [16]. The CEW Fluency Scale is a 21-item measure of common computer skills [17]. Further details about the focus groups are described in the following sections.

### Veteran Focus Groups

Veteran participants will be recruited as “expert informants”. The first round will enable veterans to describe their preferences and needs when using HIT to manage their health and exchange information. Using data collected during this phase, the research team and VA Human Factors partners will create interactive visual modeling simulations of patient-facing electronic health resources. During the second round of focus groups, participants will review the visual modeling simulations and discuss how well they represent veteran preferences and needs. Veterans will have an opportunity to provide additional input and suggest specific revisions to ensure the visual modeling simulations effectively represent their needs and preferences. Participants will also complete an adapted version of the pairwise comparison process.

### Veteran Focus Groups Round 1

Focus groups will last up to 2 hours and will be audio recorded. A moderator and facilitator will lead the focus groups, while VA operational partners and VA Human Factors representatives attend via teleconference technology. Participants will be introduced to the moderator, facilitator, and the teleconference attendees, and informed of their role and purpose for being present.

Participants will be asked questions about their preferences for using VA and non-VA electronic health resources, and specific reasons for use. Focus group questions will focus on, “The now”, resources they use (My Health***e***Vet, VetLink Kiosks, mobile phone apps, Telehealth, eBenefits, etc), resources they prefer (both VA and non-VA), tasks they commonly do, barriers and facilitators for using the tools to complete these tasks, issues with resource availability, and tools that they may or may not use if they were available, and “The future”, what they want to do, and how they want to do it. Participants will be asked to discuss: (1) their preferences for receiving information from VA and for providing information to VA to support their care; (2) their current use of electronic health resources, including which features they use, and their experiences using these resources to manage their health condition(s); and (3) their preferences for interacting with VA technology resources in the future. Sample script questions for the first round of focus groups are shown in Textbox 1.

**Sample questions for first round of focus groups.**

Preferences for exchanging information with VA:

Sharing information with your health care team is important, how would you like to be able to provide your health care team with information (for example, information that you keep track of at home)?

Use of electronic resources:

Please list all of the technology such as online resources, services, and tools (both VA and Non-VA) you currently use to manage your (condition).

Let’s talk about the tasks you do to manage your (condition), using these technologies and applications.

Probes,

-How do you use (electronic resource) to manage (condition)?

-What is the primary reason you use (electronic resource)?

-Which features of (electronic resource) are most useful to you?

Interacting with VA technology in the future:

If you were going to design the way you interact with VA using technology to send and receive information, what would it look like? Let’s draw what this system might look like together (use Post-it Pads and markers).

Can you give me an example of how VA electronic technologies could be used as an ideal system of VA electronic services that work together?

If you use the (electronic resource) to (task), would you expect the (electronic resource) to look exactly the same? If yes, how so?

Probes,

-How would you expect to see your information across tools (format)?

-How would you prefer labeling, colors, and backgrounds to be?

On smartphones and computers, there is a main page, or “dashboard” from which a user can navigate to all of their tools. How do you feel about having a dashboard of VA services and tools?

### Developing Visual Model Simulations

During the focus group, participants will be provided with pens, pads, markers, and large pieces of paper to allow them to write down their thoughts and draw out imagery that represents their preferences and needs. They will also be invited to bring any mobile devices that they use, and to share their preferred electronic resources throughout the focus group discussion. The study moderator will also transcribe notes onto large sheets of paper displayed on the wall in order to allow participants to review and refer to notes and topics throughout the focus group discussion. This method will assist the study team in guiding the discussion.

After each focus group, all notes developed during the group discussion will be immediately transcribed, and all imagery drawn by participants during the session will be photographed and saved in an electronic format. All of these assets will be transferred to the assigned Human Factors team to inform the immediate development of visual modeling simulations based on all of the data provided by veterans in the first round of focus groups.

### Developing Visual Model Simulations

Based on the data collected in round one of the focus groups, visual model simulations will be created by VA Human Factors experts using iRise version 8 (iRise, Enterprise Visualization Platform). Visual model simulations provide an effective method to rapidly create a graphic display of an electronic interface, but with limited functionality. This technique allows the user to see the graphic display (also known as wireframes), and experience it in its limited function. Focus group summaries and visual asset data (eg, drawings and sketches) gathered during the focus groups will be used to ensure that the participants’ perspectives are represented in the simulations. The study team will use focus group data to develop detailed test case scenarios and process models that will be simulated to the specified look and feel using the iRise software by the VA Human Factors team. These simulated visual models will consist of mock application screens and Web pages for various resources of interest (ie, My Health***e***Vet, Web, Mobile Health, Telehealth, and VetLink Kiosks). These models will allow veteran participants in the second round of veteran focus groups to provide feedback and make additional suggestions to further refine the visual modeling simulations until they look and function as desired.

### Veteran Focus Groups Round 2

The second round of veteran focus groups will be conducted with the same veteran participants from round one. In the second round of focus groups, participants will complete two activities: (1) provide feedback about the visual model simulations; and (2) conduct the pairwise comparison process group activity. We will provide exposure to the visual modeling simulations in group settings so that veteran focus group participants can react and provide specific feedback about these simulations and the degree to which they represent their needs and preferences. These interactive sessions will consist of a group setting, in which participants engage in a semiscripted simulation to review each of the visual modeling simulations of patient-facing platforms. As recommended by Kushniruk [18,19], participants will participate in a “brainstorming” activity, where they will talk about their experience as they access and “use” the system features. This method allows interviewers to understand what considerations veterans experience with the simulated visual prototype interfaces. Participants will have the opportunity to review the visual modeling simulations, and will be asked to vocalize thoughts, feelings, and opinions while interacting with the interface. Participants will be asked to discuss: (1) their initial thoughts about the visual model simulations; (2) their perceptions about the format and layout of the visual model simulations; and (3) the usability and ease of use of the visual model simulations. Sample script questions for the second round of focus groups are shown in Textbox 2.

**Sample questions for second round of focus groups.**

Initial responses to visual model simulations:

What are your initial thoughts about the (electronic resource simulation).

Probe,

-What are 3 things you most like/dislike about the (electronic resource simulation)?

-Does this (electronic resource simulation) reflect the feedback you gave us when we met previously?

Feedback about format and layout:

What do you like/dislike about the (colors, size, layout)?

Probe,

-How would you change the (colors, size, layout) to make (electronic resource simulation) (more useful, easier to use)?

Usability and ease of use:

Describe a scenario in which you would use this (electronic resource simulation).

How would you (navigation/task) if you wanted to (health management task) for your (health condition)?

### Usability Software to Facilitate Veteran Review of Simulations

Morae version 3.3 (Morae from TechSmith) usability software and audio recorders will be used to record and analyze revisions recommended by veteran focus group participants. This software has been utilized successfully in testing Web-based software [20,21], and allows for the live, remote observation of the users’ experiences [22]. Morae will primarily be used to record data (respondents’ reactions to the visual modeling simulations), and to then revise the simulations iteratively. Final visual model simulations will be disseminated to VA operational stakeholders to inform website redesign efforts which are currently underway.

The veteran focus group participants will also complete the pairwise comparison activity as a group to enable discussion about commonalities and differences in preferences. To accomplish this, adapted versions of the worksheets will be posted on large presentation style poster paper so that all participants can see the layout. A research team member will facilitate the pairwise comparison process with the group, allowing each participant an opportunity to select and rank electronic resources for a series of health condition management tasks. Pairwise comparisons completed by veterans will then be compared to those completed by expert panel members.

### Data Analysis

#### Expert Panel and Veteran Analytical Hierarchy Process Pairwise Comparisons

Pairwise comparisons completed by participating expert panel members and veteran focus group participants will be reviewed by the study team and then collated to identify the preferred platform for each task and function based on expressed preferences, needs, benefits, and barriers. The collation process will allow all respondent’s comparisons to be tallied to rank the usefulness of various electronic health resource platforms for completing distinct self-management and health care related tasks. The study team will also assess similarities and differences in the input provided by expert panel members versus veteran focus group participants.

#### Veteran Focus Groups

Descriptive analysis of survey and assessment data completed by veteran focus group participants will be conducted to identify sample characteristics. Qualitative data from the first round of veteran focus groups will be analyzed at two levels by three study team members. First, participant input captured on Post-it Notes will be transcribed to an electronic document. Study team members with qualitative research expertise will code the data from the Post-it Notes topographically into major domains and subdomains in order to organize and summarize the data. Using this preliminary data analysis, the team will then use this input from veterans to expand the Digital Health Matrix Model in preparation for the pairwise comparisons. Analysis of this data will also inform the creation of the visual modeling simulations.

Due to the rapid iterative nature of this project, the second level of analysis will be completed when both rounds of veteran focus groups have been completed. The audio files will be transcribed and uploaded into the qualitative data analysis software program ATLAS.ti version 7.1 (ATLAS.ti Scientific Software Development GmbH) for comprehensive analysis. Additionally, Morae recordings will be reviewed to allow the team to document notes and relevant content in alignment with the audio recorded data. This data will be compared and compiled with qualitative transcripts to collate data assets.

Once all data are collated, content analysis will be completed in two primary steps, first, we will identify domains and taxonomic structures; and second, we will evaluate coding schemas for reliability and credibility. In the first step, we will identify codes, extract meaningful statements, and identify group domains and taxonomies. Participant comments will be organized to develop codes, and codes will be merged to develop categories. Categories will be grouped into a taxonomic structure that describes the dataset. Themes identified in the preliminary analysis will be compared to those identified in subsequent transcript analyses, and results will be integrated into a final taxonomic thematic structure [23]. To complete the second step of the analysis, data samples will be extracted and coded by at least two research team members and evaluated for interrater reliability and credibility.

## Results

This paper describes a novel process and protocol for developing and implementing a mixed-methods participatory approach to evaluating and understanding veterans’ preferences and vision for an integrated system of HIT electronic health resources to support health care and self-management. Leveraging expert panel stakeholders and subject matter experts, along with veteran patients as expert consultants, we describe a participatory approach that can be used in future research to dynamically evaluate user preferences for HIT systems and tools. This approach informs the development of a more integrated and connected system of electronic health resources that will support a more holistic patient experience across multiple platforms and tools, based on a patient-centered approach to virtual care. This study is finished with recruitment, is in final stages of data collection, and the preliminary stages of data analysis.

## Discussion

### Collaborative Approaches to Development

The optimal use of health information technology to improve health care delivery and help patients become active participants in their care and self-management is essential to address patients’ ongoing health care needs [1]. HIT evaluation methods must be focused on the interactions and processes between patients, health care professionals, organizational structures, and the technology itself, to design and implement electronic health resources in ways that optimally meet user needs [1-3]. There is a distinct value in developing collaborative approaches that convene a diverse set of stakeholders to methodically define a more integrated system of electronic health resources that is synchronized with patients’ needs and preferences. A more holistic vision for “connected health” is crucial to improve resource efficiency, communication, cooperation, and collaboration across the organizational enterprise. From the end user perspective, an integrated system of electronic health resources that are standardized and synchronized will improve the end user experience, and ultimately the quality and efficiency of services provided [24]. As such, understanding the perspective of veterans, clinicians, VA operational stakeholders, and subject matter experts is vital to identifying an integrated system that can meet user needs in optimal ways [25,26].

Overall the goal of this study is to inform the VA’s vision of an integrated system of HIT electronic health resources from the shared perspective of veterans, clinicians, subject matter experts, and other key stakeholders (eg, VA operational partners). This study illustrates an innovative approach to using participatory research methods with diverse stakeholders and technology resources to create a vision for an integrated user-friendly system of HIT patient-facing resources. To our knowledge, this is one of the few published protocols that inform the development of an integrated system of HIT resources within a large health care system that serves more than 2.5 million users (Veterans and Consumers Health Informatics Office, U.S. Department of Veterans Affairs, unpublished data 2014).

### Study Limitations

Although this protocol is useful in developing valuable knowledge to inform system improvements, our study has limitations. First, although our sample size will be comparable to other qualitative mixed-methods studies [27], it is based on a small, yet representative purposively sampled group of participants and may not be generalizable to the general veteran patient population. Second, we are purposively recruiting veterans who are invested users of two or more platforms, as we feel they can provide salient in depth feedback. As such, we may miss valuable data that may represent noninvested users. Third, we are purposively including veterans with comorbid conditions because they are more likely to be consistently engaged in their health care to manage their conditions. As such, we may miss valuable data that may represent healthier participants. However, it should be noted that being in good health has been identified as a reason for not using available electronic resources [28]. Fourth, although this study includes multiple stakeholder groups, technological infrastructure capacity is not a primary focus, and thus may limit the VA’s ability to fully integrate all of the suggestions made by the participating veterans and expert panelists. However, it should be noted that the technological capacity of the current infrastructure should not limit the vision for future electronic services. Future research should inform the continued development and refinement of the VA’s vision for an integrated system of HIT resources, including both veteran patient user experiences and outcomes; and also clinical and organizational process considerations to ensure alignment with workflow processes. Both are crucial to the success of the VA’s Connected Health strategy.

Our use of mixed-methods to collect, analyze, and converge data from distinctly different sources supports the development of a product that is informed by users, clinicians, and operational Program Office representatives to identify an integrated set of electronic health resources that focus on usability and usefulness. These efforts are guided by best practices and will support a user-based design to promote integration, synchronization, and standardization across an integrated system of patient-facing platforms and tools. In alignment with VA goals and the mission of VA’s Office of Connected Health, these data will support the development and proliferation of user-friendly electronic resources that support virtual access to care that is personalized, proactive, and patient-driven to increase access, and transform the VA’s delivery of health care [8].

## Abbreviations

AHRQ: Agency for Healthcare Research and Quality
CEW: Computer-Email-Web
eHEALS: eHealth Literacy Scale
HIT: health information technology
REALM: Rapid Estimate of Adult Literacy in Medicine
VA: Department of Veterans Affairs
VHA: Veterans Health Administration

## References

[1] Nazi KM (2012). Structures and processes in health care systems. Advances in Human Aspects of Healthcare.

[2] Greenhalgh T, Robert G, Macfarlane F, Bate P, Kyriakidou O (2004). Diffusion of innovations in service organizations: Systematic review and recommendations. Milbank Q.

[3] Orlikowski WJ (2000). Using technology and constituting structures: A practice lens for studying technology in organizations. Organization Science.

[4] Nardi B, O'Day V (1999). Information ecologies: Using technology with heart: Chapter four: Information ecologies. Information ecologies: Using technology with heart.

[5] Hogan TP, Wakefield B, Nazi KM, Houston TK, Weaver FM (2011). Promoting access through complementary eHealth technologies: Recommendations for VA's home telehealth and personal health record programs. J Gen Intern Med.

[6] Nazi KM (2013). The personal health record paradox: Health care professionals' perspectives and the information ecology of personal health record systems in organizational and clinical settings. J Med Internet Res.

[7] Connected Health -- Veterans Health Administration US Department of Veterans Affairs.

[8] Department of Veterans Affairs (2014). Department of Veterans Affairs FY 2014-2020 strategic plan.

[9] Creswell Jw, Clark V (2007). Designing and conducting mixed methods research. Designing and Conducting Mixed Methods Research.

[10] Saaty TL (2008). Decision making with the analytic hierarchy process. IJSSCI.

[11] Strauss Al, Corbin JM (1998). Basics of qualitative research: Techniques and procedures for developing grounded theory.

[12] Sandelowski M (1995). Sample size in qualitative research. Res Nurs Health.

[13] Agarwal R, Khuntia J (0072). HHSA290.

[14] Haun J, Noland Dodd VJ, Graham-Pole J, Rienzo B, Donaldson P (2009). Fed Pr.

[15] Davis Tc, Long SW, Jackson RH, Mayeaux EJ, George RB, Murphy PW, Crouch MA (1993). Rapid estimate of adult literacy in medicine: A shortened screening instrument. Fam Med.

[16] Norman CD, Skinner HA (2006). eHEALS: The eHealth literacy scale. J Med Internet Res.

[17] Bunz U (2004). The computer-email-web (CEW) fluency scale-development and validation. International Journal of Human-Computer Interaction.

[18] Kushniruk AW, Patel VL (1995). Cognitive computer-based video analysis: Its application in assessing the usability of medical systems. Medinfo.

[19] Kushniruk AW (2001). Analysis of complex decision-making processes in health care: Cognitive approaches to health informatics. Journal of Biomedical Informatics.

[20] Yen PY, Bakken S (2009). Usability testing of a web-based tool for managing open shifts on nursing units. Stud Health Technol Inform.

[21] Choi J, Bakken S (2006). Heuristic evaluation of a web-based educational resource for low literacy NICU parents. Stud Health Technol Inform.

[22] Johnston Lg, Malekinejad M, Kendall C, Iuppa IM, Rutherford GW (2008). Implementation challenges to using respondent-driven sampling methodology for HIV biological and behavioral surveillance: Field experiences in international settings. AIDS Behav.

[23] Corbin Jm, Strauss AL (2007). Basics of qualitative research: Techniques and procedures for developing grounded theory.

[24] Bodenheimer T California Health Care Foundation.

[25] Kaplan B (2001). Evaluating informatics applications--some alternative approaches: Theory, social interactionism, and call for methodological pluralism. Int J Med Inform.

[26] Kaplan B, Harris-Salamone KD (2009). Health IT success and failure: Recommendations from literature and an AMIA workshop. J Am Med Inform Assoc JAMIA.

[27] Guest G, Bunce AJ, Johnson L (2006). How many interviews are enough?: An experiment with data saturation and variability. Field Methods.

[28] Haun JN, Lind JD, Shimada SL, Simon SR (2014). Evaluating secure messaging from the veteran perspective: Informing the adoption and sustained use of a patient-driven communication platform. Annals of Anthropological Practice.

